# Psychological Inflexibility and Suicidal Ideation in Pregnant Women: A Cross-Sectional Multiple Mediation Analysis of Depressive Symptoms, Insomnia, and Generalized Anxiety

**DOI:** 10.3390/ejihpe16100151

**Published:** 2026-09-27

**Authors:** Jonatan Baños-Chaparro, Juan Artica-Martinez, Tomás Caycho-Rodríguez

**Affiliations:** 1 Programa Académico de Psicología, Facultad de Ciencias de la Salud, Universidad Privada Norbert Wiener, Lima 15046, Peru; 2 Departamento de Psicología, Instituto Nacional Materno Perinatal, Lima 15046, Peru; jartica@iemp.gob.pe; 3 Facultad de Psicología, Universidad Científica del Sur, Lima 15046, Peru; tcaycho@cientifica.edu.pe

**Keywords:** psychological inflexibility, suicidal ideation, pregnancy, depression, anxiety, insomnia

## Abstract

**Introduction**: Suicidal ideation during pregnancy is an important public health concern. Psychological inflexibility may be associated with emotional and sleep-related symptoms linked to suicidal ideation; however, these patterns of association remain insufficiently understood in pregnant women. **Objective**: The objective was to examine the relationship between psychological inflexibility and suicidal ideation in pregnant women by evaluating the multiple mediating roles of depressive symptoms, insomnia, and generalized anxiety. **Method**: A quantitative, cross-sectional associative study using latent variables was conducted with 962 pregnant women receiving care at the National Maternal and Perinatal Institute (INMP), located in Cercado de Lima, Lima, Peru, between October and December 2025. Participants completed the Acceptance and Action Questionnaire-II (AAQ-II), Frequency of Suicidal Ideation Inventory (FSII), Athens Insomnia Scale (AIS), Patient Health Questionnaire-2 (PHQ-2), and Generalized Anxiety Disorder-2 (GAD-2). Structural equation modeling (SEM) was performed using R version 4.5.1 within the RStudio Version 4.5.1 environment. **Results**: The proposed model showed a good fit, scaled χ^2^(179) = 506.334, *p* < 0.001, robust CFI = 0.968, robust TLI = 0.963, robust RMSEA = 0.052, 90% CI [0.046, 0.057], and SRMR = 0.030. Psychological inflexibility showed a significant direct association with suicidal ideation (β = 0.246, *p* < 0.001) and significant indirect associations through depressive symptoms (β = 0.245, *p* < 0.001) and insomnia (β = 0.030, *p* = 0.007), whereas the indirect association through generalized anxiety was not significant (β = 0.007, *p* = 0.749). The total indirect association was significant (β = 0.282, *p* < 0.001), while the direct association remained statistically significant after inclusion of the mediators. **Conclusions**: Psychological inflexibility was associated with suicidal ideation among pregnant women, with depressive symptoms and insomnia showing significant indirect associations. These cross-sectional findings identify relevant correlates of suicidal ideation that warrant further longitudinal investigation.

## 1. Introduction

Suicidal ideation encompasses thoughts, images, or wishes related to one’s own death or to the intention to end one’s life. This phenomenon may occur along a continuum ranging from passive thoughts, such as wishing not to continue living, to more active thoughts involving the consideration of methods or the development of suicide plans (Baños-Chaparro, 2022). In the perinatal context, its assessment is particularly relevant because it represents a proximal indicator of suicide risk and enables the timely identification of women who may require care before the onset of self-harming behaviors or suicide attempts (Legazpi et al., 2022). International epidemiological evidence indicates that suicidal ideation during pregnancy constitutes a relevant maternal mental health concern. Its prevalence may be similar to, or even higher than, that observed in the general population, although estimates vary widely depending on the country, sample characteristics, assessment instrument, and reference period considered (Faisal-Cury et al., 2022; Gelaye et al., 2016; Legazpi et al., 2022). A review including studies conducted in several countries, including Peru, found that prevalence rates among pregnant women ranged approximately from 5% to 20%, with even higher percentages reported in certain contexts of vulnerability (Gelaye et al., 2016). Consistently, another international review reported estimates ranging from 2.7% to 18% (Legazpi et al., 2022). In Latin America, the available evidence remains limited and shows substantial methodological differences in assessment approaches. Nevertheless, existing studies reveal clinically relevant levels of suicidal ideation among both adult women and particularly vulnerable groups, including adolescents (Faisal-Cury et al., 2022; Soto-Chavarría et al., 2023). In Peru, a study involving 1517 pregnant women receiving care in Lima identified prevalence rates of suicidal ideation ranging from 8.8% to 15.8%. These findings also showed that the estimated magnitude of the phenomenon may vary according to the instrument used and the time frame considered for its assessment (Zhong et al., 2015).

Mental health during pregnancy is essential for women’s well-being and for appropriate adaptation to the physical, emotional, family, and social changes inherent to this stage of life. Pregnant women may experience depressive symptoms, anxiety, sleep disturbances, stress, concerns related to fetal health, financial difficulties, intimate partner violence, and limited social support (Anggraini et al., 2022; Dadi et al., 2024; Gelaye et al., 2017). Among these factors, psychological inflexibility constitutes a particularly relevant transdiagnostic process. Within the Acceptance and Commitment Therapy model, psychological inflexibility is expressed as a tendency to respond rigidly to unpleasant thoughts, emotions, or sensations, allowing these internal experiences to control behavior and interfere with actions guided by personal values (Macri & Rogge, 2024). This pattern may increase hopelessness, isolation, self-criticism, and the perception that no alternatives are available for coping with distress (Macri & Rogge, 2024). Longitudinal studies conducted in non-perinatal populations have shown that psychological inflexibility predicts subsequent increases in suicidal ideation even after controlling for psychological distress, baseline suicidal ideation, and other psychological risk factors. These findings support its consideration as a potentially modifiable target for prevention, although this relationship needs to be examined specifically among pregnant women (DeBeer et al., 2017; Krafft et al., 2018).

Depressive symptoms, insomnia, and generalized anxiety may represent distinct patterns of indirect association between psychological inflexibility and suicidal ideation during pregnancy (Djatche Miafo et al., 2023; Rafihi-Ferreira et al., 2024; Legazpi et al., 2022). First, experiential avoidance, cognitive fusion, and difficulty maintaining valued behaviors may be associated with depressive symptoms such as persistent sadness, loss of interest, guilt, hopelessness, and reduced problem-solving capacity; these states have been associated with greater suicidal ideation and perceptions of death as a means of escaping suffering (Djatche Miafo et al., 2023; Fernández-Rodríguez et al., 2022). Second, psychological inflexibility may be associated with insomnia, including in the context of repetitive worry, difficulty accepting intrusive thoughts, and increased cognitive arousal at night (Rafihi-Ferreira et al., 2024). Sleep deprivation or disruption has been associated with difficulties in emotional regulation, greater impulsivity, and reduced ability to cope with stress (Mastin-Purcell et al., 2025). Indeed, among pregnant women in Peru, poor sleep quality was associated with greater odds of suicidal ideation even after controlling for depressive symptoms (Gelaye et al., 2017). Third, generalized anxiety may represent another relevant correlate in this pattern of associations, as fusion with anticipated threats and the perceived uncontrollability of worry may sustain a persistent state of hypervigilance, distress, and psychological exhaustion (Fernández-Rodríguez et al., 2022). Although these mediators are related to one another, a parallel multiple mediation model makes it possible to estimate whether each contributes a specific indirect effect on suicidal ideation while partially controlling for the variance shared with the other mediators. Therefore, the proposed model does not assume that depression, insomnia, and anxiety are mutually exclusive or necessarily sequential processes, but rather concurrent and potentially complementary patterns of association.

Despite the available international and Peruvian evidence on suicidal ideation during pregnancy, the patterns of association linking psychological inflexibility with suicidal ideation and the specific indirect associations involving depressive symptoms, insomnia, and generalized anxiety remain insufficiently examined in pregnant women, particularly in the Peruvian context. Examining these variables simultaneously may provide a more specific understanding of how these interrelated psychological factors are associated with suicidal ideation in this population. In this context, investigating the patterns of association involving suicidal ideation among pregnant women is important for strengthening early detection and guiding psychological interventions targeting modifiable processes (Faisal-Cury et al., 2022; Gelaye et al., 2016; Legazpi et al., 2022; Soto-Chavarría et al., 2023). The present study aimed to examine the relationship between psychological inflexibility and suicidal ideation in pregnant women by evaluating the multiple mediating roles of depressive symptoms, insomnia, and generalized anxiety. Specifically, the study sought to determine: (a) the direct association between psychological inflexibility and suicidal ideation; (b) the associations between psychological inflexibility and depressive symptoms, insomnia, and generalized anxiety; (c) the relationships between these three mental health indicators and suicidal ideation; and (d) the specific and combined indirect effects of these variables on the relationship between psychological inflexibility and suicidal ideation. It was hypothesized that greater psychological inflexibility would be associated with higher levels of suicidal ideation. Psychological inflexibility was also expected to be positively associated with depressive symptoms, insomnia, and generalized anxiety, and each of these variables was hypothesized to positively mediate the relationship between psychological inflexibility and suicidal ideation.

## 2. Materials and Methods

### 2.1. Design

The study was conducted using an associative strategy with an explanatory design based on a mediation model including both latent and observed variables (Ato et al., 2013). It also followed a quantitative, basic, and cross-sectional approach.

### 2.2. Participants

A total of 962 Peruvian pregnant women participated in the study. They were selected through non-probabilistic convenience sampling and met the following inclusion criteria: (a) being 18 years of age or older, (b) residing in Metropolitan Lima, (c) receiving perinatal care at the National Maternal and Perinatal Institute, and (d) being in the first, second, or third trimester of pregnancy. The exclusion criteria were (a) diagnosis of an intellectual or neurological developmental disorder that prevented completion of the survey, (b) incomplete responses in the sociodemographic section or in the psychological instruments, and (c) voluntary withdrawal from the survey. The sample size was calculated using Soper’s version 4.0 calculator, based on a structural model comprising five latent variables and 21 observed variables (Soper, 2026). The calculation considered an anticipated effect size of 0.20, statistical power of 0.99, and a *p*-value of 0.05, yielding a minimum recommended sample size of 743 participants. The final sample size was larger than the minimum recommended sample size.

### 2.3. Measures

**Sociodemographic Information.** A questionnaire section developed specifically for the purposes of this study was used to collect participants’ sociodemographic and pregnancy-related data, including age, employment, marital status, area of residence, educational level, gestational stage, planned pregnancy, perceived partner rejection of the current pregnancy, abortion history, current intention to terminate the pregnancy, and attitude toward pregnancy. The section also included separate yes/no questions regarding suicidal ideation and suicide attempts during the current pregnancy. These variables were used for descriptive purposes and were distinct from the Frequency of Suicidal Ideation Inventory (FSII; Chang & Chang, 2016), which was included as the measure of suicidal ideation in the structural model and has shown psychometric support in Peruvian pregnant women (Baños-Chaparro et al., 2026). Perceived partner rejection referred to the pregnant woman’s perception of rejection by her partner toward the current pregnancy and was assessed according to its reported frequency.

**Acceptance and Action Questionnaire-II (AAQ-II).** This questionnaire consists of 7 items measuring psychological inflexibility. Each item is rated on a 7-point scale. Total scores range from 7 to 49, with higher scores reflecting greater psychological inflexibility. The Spanish version developed by Ruiz et al. (2016) was used. Evidence supporting its use in Peru has been reported among university students from Lima by Valencia and Falcón (2022). In the present sample of pregnant women, model-based internal consistency was satisfactory (ω = 0.92).

**Frequency of Suicidal Ideation Inventory (FSII).** This instrument measures the frequency of suicidal thoughts during the past year through five items (Chang & Chang, 2016). Because the FSII assesses suicidal ideation over the previous 12 months, scores may reflect suicidal thoughts occurring either during the current pregnancy or before its onset, depending on gestational age. Accordingly, in the present study, FSII scores were interpreted as past-year suicidal ideation reported by pregnant women rather than as ideation occurring exclusively during pregnancy. Each item is rated on a Likert scale from 1 (never) to 5 (almost always). The total score ranges from 5 to 25, with higher values reflecting a greater frequency of suicidal ideation. Importantly, the version used in the present study has been specifically evaluated in pregnant women and showed evidence of validity and high internal consistency (α = 0.95) (Baños-Chaparro et al., 2026). In the present sample, model-based internal consistency was also high (ω = 0.95).

**Athens Insomnia Scale (AIS).** This scale consists of five items that assess the presence of insomnia symptoms during the past month, considering a frequency of at least three times per week. Responses are recorded on a Likert scale from 0 to 3 (Soldatos et al., 2000). Total scores range from 0 to 15, where higher scores indicate greater severity of insomnia symptoms. Psychometric evidence for the AIS has been reported in Peruvian adults, with adequate internal consistency in the validation study (α = 0.89) (Baños-Chaparro et al., 2021). In the present sample of pregnant women, model-based internal consistency was satisfactory (ω = 0.89).

**Patient Health Questionnaire-2 (PHQ-2).** This is a brief instrument with two items that measure depressive symptoms during the past two weeks. Each item is rated on a 4-point scale from 0 (not at all) to 3 (nearly every day) (Kroenke et al., 2003). Total scores range from 0 to 6, where higher scores reflect greater severity of depressive symptoms. Evidence regarding the diagnostic performance of the PHQ-2 has been reported in a Peruvian hospital population (Villarreal-Zegarra et al., 2023). Although this validation was not conducted specifically in pregnant women, the instrument was retained because of its established use as a brief measure of depressive symptoms and its previous evaluation in the Peruvian context. In the present sample, model-based internal consistency was adequate (ω = 0.80).

**Generalized Anxiety Disorder-2 (GAD-2).** This is a brief instrument composed of two items that measure generalized anxiety symptoms during the past two weeks. Each item is rated on a 4-point scale from 0 (not at all) to 3 (nearly every day) (Kroenke et al., 2007). Total scores range from 0 to 6, with higher scores indicating greater levels of generalized anxiety. Psychometric evidence for the GAD-2 has been reported among Peruvian university students, with adequate internal consistency in the validation study (α = 0.86) (Franco-Jimenez & Nuñez-Magallanes, 2022). Although pregnancy-specific Peruvian validation evidence is currently limited, the instrument was used because of its brevity, established psychometric evidence, and previous evaluation in the Peruvian context. In the present sample, model-based internal consistency was adequate (ω = 0.87).

### 2.4. Procedure

Data were collected in person at the National Maternal and Perinatal Institute (INMP), located in Cercado de Lima, between October and December 2025. Four psychologists from the institution, previously trained in survey administration and ethical research procedures, administered the instruments. Potential participants were approached in the INMP perinatal care services. The questionnaires were completed individually, in person, in a setting within the INMP that ensured participants’ privacy and comfort and took approximately 10 to 15 min to complete.

Given the sensitive nature of the assessment of suicidal ideation, a predefined suicide risk-management protocol was available throughout data collection. The psychologists were also trained to identify active suicidal ideation and signs of clinically significant psychological distress and to activate the protocol when necessary. Participants who reported suicidal ideation without indicators of immediate risk received preventive information about available mental health resources and support services. Participants identified as presenting active risk were not left unattended, received immediate psychological support and evaluation by the attending psychologist, and were referred through the established institutional pathway to the mental health services of the INMP for specialized assessment and care. Although 53 participants reported suicidal ideation during the assessment, four met the criteria for immediate intervention because they presented active suicidal ideation or significant psychological distress at the time of data collection. Because the survey was anonymous and no contact information was linked to the research database, follow-up was not conducted by the research team; however, participants referred for specialized care entered the institutional mental health pathway, where subsequent assessment and follow-up could be provided according to clinical procedures.

Before participating, the women were informed about the objectives of the study, its exclusively academic purpose, the voluntary nature of their participation, the data management procedures, and the safeguards for anonymity and confidentiality. Written informed consent was obtained in person from all participants, in accordance with ethical standards for research involving human subjects. Participants were also informed that they could decline to answer any question or discontinue the assessment at any time without affecting their clinical care or access to health services.

### 2.5. Ethical Considerations

The study adhered to the ethical guidelines of the American Psychological Association (APA) and the Code of Ethics of the Peruvian College of Psychologists (CPsP), as described in Chapter Six on good research practices (Colegio de Psicólogos del Perú, 2024; Knapp & VandeCreek, 2003). All pregnant women provided informed consent prior to participation. The survey was anonymous and voluntary, and confidentiality of the data was guaranteed. The study was reviewed and approved by the Ethics Committee of Universidad Privada Norbert Wiener under registration number A0019-2025-CIEIC-UPNW (approved on 4 June 2025), as well as by the Ethics Committee of the National Maternal Perinatal Institute under registration N°0171-2025-DG-N°0118-OEAIDE-INMP (approved on 29 September 2025).

### 2.6. Data Analysis

All statistical analyses were conducted in R version 4.5.1 (R Core Team, 2025) using RStudio as the integrated development environment. In the first phase, descriptive analyses of the psychological variables were performed, including measures of central tendency and dispersion such as the mean and standard deviation. Associations between variables were examined using Pearson’s correlation coefficient, with effect sizes interpreted as small (r = 0.10), medium (r = 0.20), large (r = 0.30), and very large (r = 0.40) (Funder & Ozer, 2019/2020). To account for multiple pairwise correlation tests, *p*-values were adjusted using the Holm method.

The measurement component of the SEM comprised five latent variables represented by 21 item-level observed indicators. Psychological inflexibility was specified using the seven AAQ-II items, depressive symptoms using the two PHQ-2 items, generalized anxiety using the two GAD-2 items, insomnia using the five AIS items, and suicidal ideation using the five FSII items (Chang & Chang, 2016; Baños-Chaparro et al., 2026). For each latent variable, the loading of the first indicator was fixed to 1.0 to set the scale of the factor, while the remaining factor loadings were freely estimated. Thus, the PHQ-2 and GAD-2 were modeled as two-indicator latent variables rather than as observed total scores.

The structural model was specified a priori according to the study objectives and theoretical framework. No sociodemographic or obstetric covariates were included because the purpose of the analysis was to examine the prespecified pattern of associations among psychological inflexibility, the three proposed mediators, and suicidal ideation, rather than to estimate a comprehensive covariate-adjusted model.

In the second phase, the distributional characteristics of the 21 item-level indicators were examined through skewness and kurtosis, and multivariate normality was assessed using Mardia’s test. Several indicators, particularly those assessing suicidal ideation, showed substantial departures from normality, and Mardia’s test indicated significant multivariate non-normality. Considering the observed departures from multivariate normality, the mediation model was estimated using robust maximum likelihood (MLR), which provides robust standard errors and a scaled chi-square statistic. Although the indicators comprised four to seven ordered response categories, they were treated as approximately continuous variables within the robust ML framework (Muthén & Muthén, 2017; Rhemtulla et al., 2012; Li, 2016). The measurement component comprised five latent variables represented by 21 observed item-level indicators: psychological inflexibility by the seven AAQ-II items, depressive symptoms by the two PHQ-2 items, generalized anxiety by the two GAD-2 items, insomnia by the five AIS items, and suicidal ideation by the five FSII items (Chang & Chang, 2016; Baños-Chaparro et al., 2026). The loading of the first indicator of each latent variable was fixed to 1.0 to establish the metric of the factor, while the remaining factor loadings were freely estimated. Model fit was evaluated using the robust Comparative Fit Index (CFI > 0.90), robust Tucker–Lewis Index (TLI > 0.90), robust Root Mean Square Error of Approximation (RMSEA < 0.08) with its 90% confidence interval, and the Standardized Root Mean Square Residual (SRMR < 0.08) (Jordan, 2021). At the local level, standardized regression coefficients (β) were examined, with effect sizes interpreted as minimal (β = 0.10), moderate (β = 0.50), and strong (β = 0.80) (Ferguson, 2016). Direct, specific indirect, total indirect, and total effects were estimated, and statistical significance was established at *p* < 0.05.

The final analytic dataset contained no missing values for the variables included in the analyses; therefore, no imputation or model-based missing-data procedure was required. Model convergence and admissibility were also examined. The model converged normally after 59 iterations, with no negative residual variances or standardized correlations outside the admissible range.

## 3. Results

### 3.1. Sociodemographic Characteristics of the Participants

Table 1 summarizes the participants’ sociodemographic profile. Participants had a mean age of 30.97 years (SD = 6.68), with ages ranging from 18 to 48 years. Most women were not currently employed (66.6%) and were single (63.7%), whereas 34.3% were married. The majority lived in urban areas (85.3%) and had completed secondary education (34.8%); technical education was the next most frequent educational level (17.2%). With respect to pregnancy, 42.6% of participants were in their third trimester, 37.8% were in the second trimester, and 19.5% were in the first trimester. Half of the pregnancies were planned (50.2%), and 76.5% of participants reported never perceiving rejection from their partner regarding the current pregnancy. Suicidal ideation was reported by 5.5% of the sample, while 1.1% indicated a history of suicide attempts. Regarding abortion history, 53.2% had not experienced any abortion, although 37.6% reported a previous miscarriage. Finally, most participants reported no current intention to terminate the pregnancy (96.6%), and 52.3% expressed a positive attitude toward their pregnancy.

### 3.2. Descriptive Analysis

Descriptive statistics indicated that psychological inflexibility had the highest mean and standard deviation (M = 18.01, SD = 8.84), followed by suicidal ideation (M = 6.28, SD = 3.10), insomnia (M = 4.83, SD = 3.68), generalized anxiety (M = 1.40, SD = 1.66), and depressive symptoms (M = 1.15, SD = 1.56). Figure 1 presents the correlation matrix among the psychological variables. All variables were positively and statistically significantly correlated, with effect sizes ranging from small to moderate and large.

### 3.3. Measurement Component

The measurement component showed satisfactory factor loadings across the five latent constructs. Standardized factor loadings ranged from 0.697 to 0.925, and all freely estimated loadings were statistically significant (*p* < 0.001). Specifically, loadings ranged from 0.706 to 0.827 for psychological inflexibility, from 0.701 to 0.925 for depressive symptoms, from 0.854 to 0.892 for generalized anxiety, from 0.697 to 0.868 for insomnia, and from 0.847 to 0.921 for suicidal ideation. The complete factor-loading estimates are presented in Table 2.

### 3.4. Mediation Analysis

A multiple mediation model was estimated in which psychological inflexibility showed both direct and indirect associations with suicidal ideation, with specific indirect associations observed through depressive symptoms, generalized anxiety, and insomnia. The model demonstrated a good fit to the data, scaled χ^2^(179) = 506.334, *p* < 0.001, robust CFI = 0.968, robust TLI = 0.963, robust RMSEA = 0.052, 90% CI [0.046, 0.057], and SRMR = 0.030 (Figure 2).

Psychological inflexibility was positively associated with depressive symptoms (β = 0.507, *p* < 0.001), generalized anxiety (β = 0.459, *p* < 0.001), and insomnia (β = 0.303, *p* < 0.001). In turn, depressive symptoms (β = 0.483, *p* < 0.001) and insomnia (β = 0.098, *p* = 0.003) were significantly associated with suicidal ideation, whereas generalized anxiety was not (β = 0.016, *p* = 0.749).

Regarding the indirect associations (Table 3), psychological inflexibility showed a significant indirect association with suicidal ideation through depressive symptoms (β = 0.245, *p* < 0.001) and insomnia (β = 0.030, *p* = 0.007). In contrast, the indirect association through generalized anxiety was not significant (β = 0.007, *p* = 0.749). The total indirect effect was significant (β = 0.282, *p* < 0.001), as was the total effect of psychological inflexibility on suicidal ideation (β = 0.528, *p* < 0.001). The direct association remained significant after inclusion of the mediators (β = 0.246, *p* < 0.001), alongside a significant total indirect association.

## 4. Discussion

Pregnancy involves physical, hormonal, emotional, and social changes that may increase psychological vulnerability. Concerns about fetal health, childbirth, motherhood, financial circumstances, and family support may generate distress, particularly when pregnant women exhibit psychological inflexibility, that is, difficulties adapting to circumstances and relating flexibly to unpleasant thoughts and emotions (Legazpi et al., 2022). In this context, depressive symptoms, generalized anxiety, and insomnia may show indirect associations between psychological inflexibility and suicidal ideation. Depressive symptoms may include hopelessness, loss of interest, and feelings of inadequacy; anxiety may manifest as persistent concerns about pregnancy and motherhood; and insomnia may intensify fatigue, irritability, and difficulties in emotion regulation. Therefore, the aim of the study was to analyze the mediating role of these three variables among pregnant women, a population in which suicidal ideation represents a relevant maternal health concern (Gelaye et al., 2017; Legazpi et al., 2022; Zhong et al., 2015).

The results showed that psychological inflexibility was positively associated with depressive symptoms, generalized anxiety, and insomnia, supporting its conceptualization as a transdiagnostic process associated with different manifestations of psychological distress (Fernández-Rodríguez et al., 2022; Macri & Rogge, 2024). However, the indirect associations showed different patterns. The largest indirect association between psychological inflexibility and suicidal ideation was observed through depressive symptoms. One possible explanation is that psychologically inflexible pregnant women may become fused with negative thoughts concerning their ability to cope with motherhood, interpret their current difficulties as permanent, and reduce their engagement in rewarding activities or sources of support (Djatche Miafo et al., 2023; Fernández-Rodríguez et al., 2022). This pattern may be associated with hopelessness, isolation, perceptions of inadequacy, and suicidal ideation. This finding is consistent with studies identifying depressive symptoms as one of the main factors associated with suicidal ideation among pregnant women (Faisal-Cury et al., 2022; Gelaye et al., 2016; Legazpi et al., 2022).

A significant indirect association through insomnia was also observed, although its magnitude was smaller. During pregnancy, physical discomfort, fetal movements, frequent urination, pain, reflux, and concerns about childbirth may interfere with sleep; however, psychological inflexibility may be associated with these sleep difficulties, including in the context of nighttime rumination, efforts to control thoughts, and difficulties adopting a flexible attitude toward distress (Gelaye et al., 2017; Mastin-Purcell et al., 2025). Sleep deprivation or fragmentation has been associated with poorer emotion regulation, greater irritability, more negative thoughts, reduced problem-solving capacity, and suicidal ideation (Mastin-Purcell et al., 2025). This finding is consistent with the previously observed association between poor sleep quality, depression, and suicidal ideation among pregnant women in Peru (Gelaye et al., 2017), as well as with studies linking psychological inflexibility to insomnia and pre-sleep cognitive arousal (Mastin-Purcell et al., 2025; Rafihi-Ferreira et al., 2024). In contrast, although psychological inflexibility was significantly associated with generalized anxiety, the latter was not independently associated with suicidal ideation and did not show a significant indirect effect. This finding does not imply that anxiety is irrelevant; rather, its association may overlap with that of depressive symptoms and insomnia (Fernández-Rodríguez et al., 2022). It is also possible that anxious worry may not show an independent association with suicidal ideation after accounting for depressive symptoms and insomnia (Legazpi et al., 2022). Furthermore, a general measure of anxiety may fail to capture specifically perinatal fears, such as fear of childbirth, obstetric complications, fetal loss, or not adequately fulfilling the maternal role (Anggraini et al., 2022).

At the theoretical level, the findings are consistent with considering psychological inflexibility as a relevant correlate of mental health during pregnancy, given its simultaneous associations with depressive symptoms, generalized anxiety, and insomnia. However, the results also indicate that these variables did not show the same pattern of indirect association with suicidal ideation. The largest indirect association was observed through depressive symptoms, followed by insomnia, whereas generalized anxiety did not show a specific indirect contribution. This pattern suggests that the association between psychological inflexibility and suicidal ideation was stronger through depressive symptoms and, to a lesser extent, insomnia (Djatche Miafo et al., 2023; Mastin-Purcell et al., 2025). Moreover, the fact that the direct effect remained significant after the mediators were included indicates partial mediation and suggests that other unmeasured psychological, social, or obstetric variables may also be associated with this relationship, such as hopelessness, psychological entrapment, perceived burdensomeness, self-criticism, loneliness, intimate partner violence, or lack of social support (Legazpi et al., 2022). In practical terms, the findings suggest that psychological inflexibility and sleep quality may warrant further consideration as potential components of broader prenatal mental health assessment, alongside established assessment of depressive symptoms, anxiety, and suicide risk. These findings may help identify variables for further investigation rather than establish specific clinical targets. Future longitudinal and intervention studies should examine whether psychological flexibility, depressive symptoms, and sleep disturbances prospectively contribute to suicidal ideation and whether interventions targeting these factors are effective in reducing suicidal ideation among pregnant women. If supported by such evidence, approaches addressing psychological flexibility could be evaluated alongside established interventions for depressive symptoms and sleep problems. More broadly, strengthening the capacity of obstetricians, psychologists, physicians, and nurses to identify warning signs, provide timely referrals, and establish follow-up procedures may contribute to improved perinatal mental health care.

Among the main strengths of the study is its analysis of a particularly vulnerable population that remains understudied in the Latin American context: pregnant women. In addition, the simultaneous assessment of three mediators made it possible to compare their specific contributions and determine that depressive symptoms, insomnia, and generalized anxiety showed different patterns of indirect association with suicidal ideation. Another strength was the separate estimation of direct, indirect, and total effects, which made it possible to characterize the specific pattern and magnitude of the direct and indirect associations. Identify potentially modifiable variables that warrant further examination in longitudinal and intervention studies. However, the findings should be interpreted in light of several limitations. First, non-probability sampling restricts the representativeness of the sample and limits the generalizability of the findings to all pregnant women in Peru; therefore, future research should use probability-based or multicenter sampling and include participants from private healthcare facilities, different regions of the country, and rural areas. Second, the cross-sectional nature of the data precludes establishing temporal precedence and causal relationships among the variables. Accordingly, the observed indirect associations should be interpreted as statistically consistent with the proposed mediation model rather than as evidence of established causal mechanisms. Future studies should employ longitudinal designs, assessing psychological inflexibility early in pregnancy, emotional symptoms and insomnia at subsequent time points, and suicidal ideation during the third trimester or postpartum period. Third, the exclusive use of self-report measures may have increased common method bias, social desirability bias, and the concealment of suicidal thoughts because of fear of stigma or possible clinical consequences. Future research should therefore combine questionnaires with structured clinical interviews. Fourth, although all instruments had previous psychometric evidence in Spanish-speaking or Peruvian populations and showed adequate model-based reliability in the present sample, pregnancy-specific Peruvian validation evidence was not available for all measures. Therefore, further studies should examine their measurement properties specifically in pregnant populations. In addition, the FSII assesses suicidal ideation over the previous 12 months (Chang & Chang, 2016); therefore, particularly among women in earlier stages of pregnancy, reported suicidal ideation may have preceded the current pregnancy. Consequently, the findings should be interpreted as referring to past-year suicidal ideation among pregnant women rather than exclusively to suicidal ideation occurring during pregnancy. Finally, the structural model was intentionally restricted to the variables specified in the theoretical mediation model and did not adjust for additional sociodemographic, psychological, or obstetric characteristics. Consequently, residual confounding by unmeasured or unmodeled factors cannot be ruled out, and the estimated associations should not be interpreted as covariate-adjusted effects. Future studies should examine the robustness of these associations after considering theoretically relevant factors such as hopelessness, entrapment, perceived burdensomeness, intimate partner violence, social support, history of suicide attempts, unplanned pregnancy, obstetric complications, financial difficulties, and history of mental disorders.

## 5. Conclusions

Psychological inflexibility was positively associated with suicidal ideation among pregnant women, both directly and through statistically significant indirect associations. The largest indirect association was observed through depressive symptoms, whereas the indirect association through insomnia was significant but smaller. In contrast, no significant indirect association was observed through generalized anxiety when the three candidate mediators were analyzed simultaneously. The persistence of a significant direct association after accounting for the mediators indicates a pattern statistically consistent with partial mediation and suggests that other psychological, social, or obstetric variables not included in the model may also be relevant to the association between psychological inflexibility and suicidal ideation.

Taken together, these findings indicate that suicidal ideation among pregnant women is associated not only with depressive symptoms and sleep disturbances but also with psychological inflexibility. Given the cross-sectional nature of the study, these associations should not be interpreted as evidence of temporal or causal mechanisms. Rather, the results identify psychological inflexibility, depressive symptoms, and insomnia as relevant correlates of suicidal ideation in pregnant women. These findings may inform future longitudinal and intervention studies examining the temporal relationships among these variables and whether their assessment may contribute to strengthening early detection and mental health care during pregnancy in Peru.

## Figures and Tables

**Figure 1:**
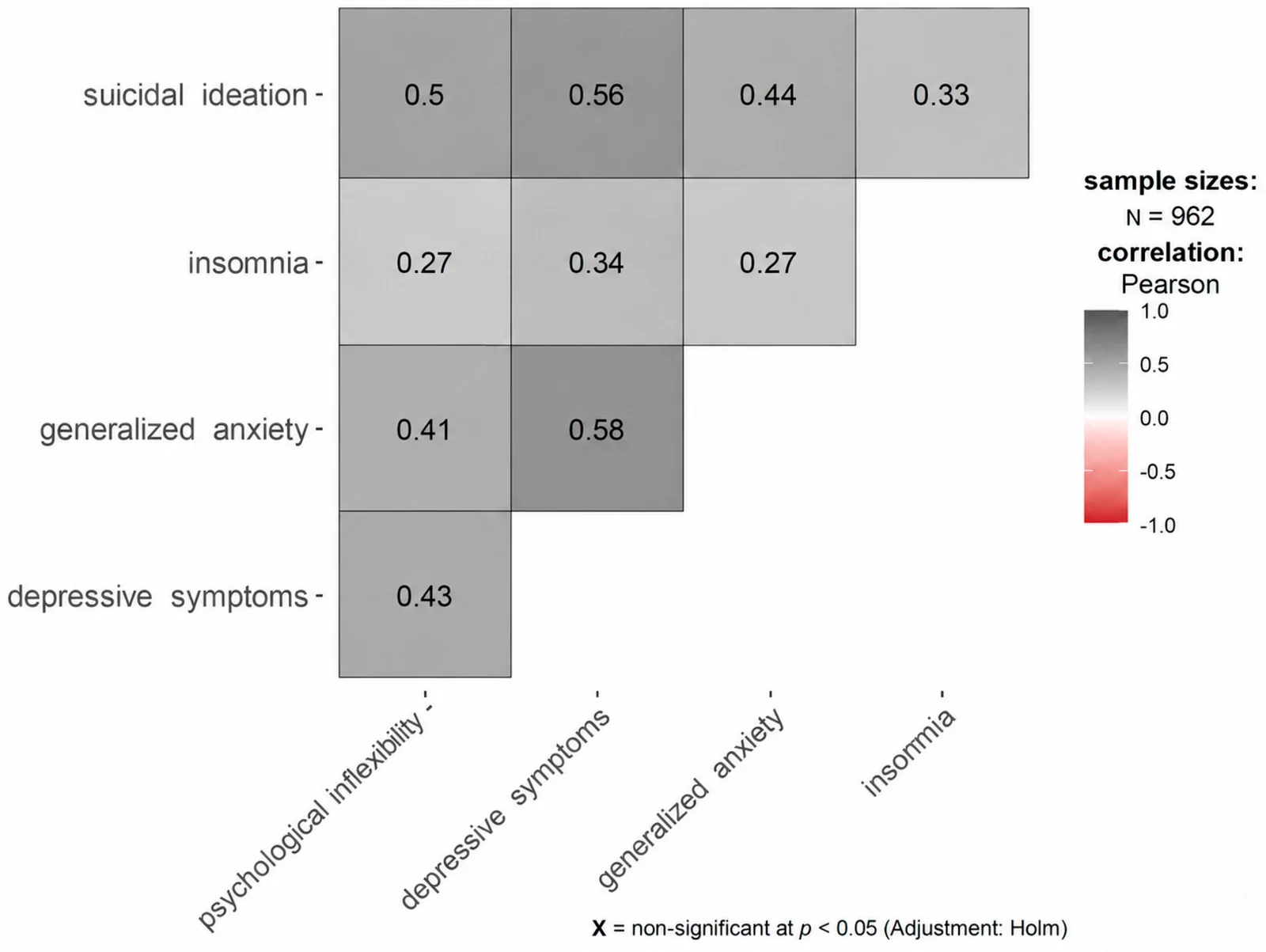
Correlation matrix among psychological inflexibility, insomnia, generalized anxiety, depressive symptoms, and suicidal ideation in pregnant women.

**Figure 2:**
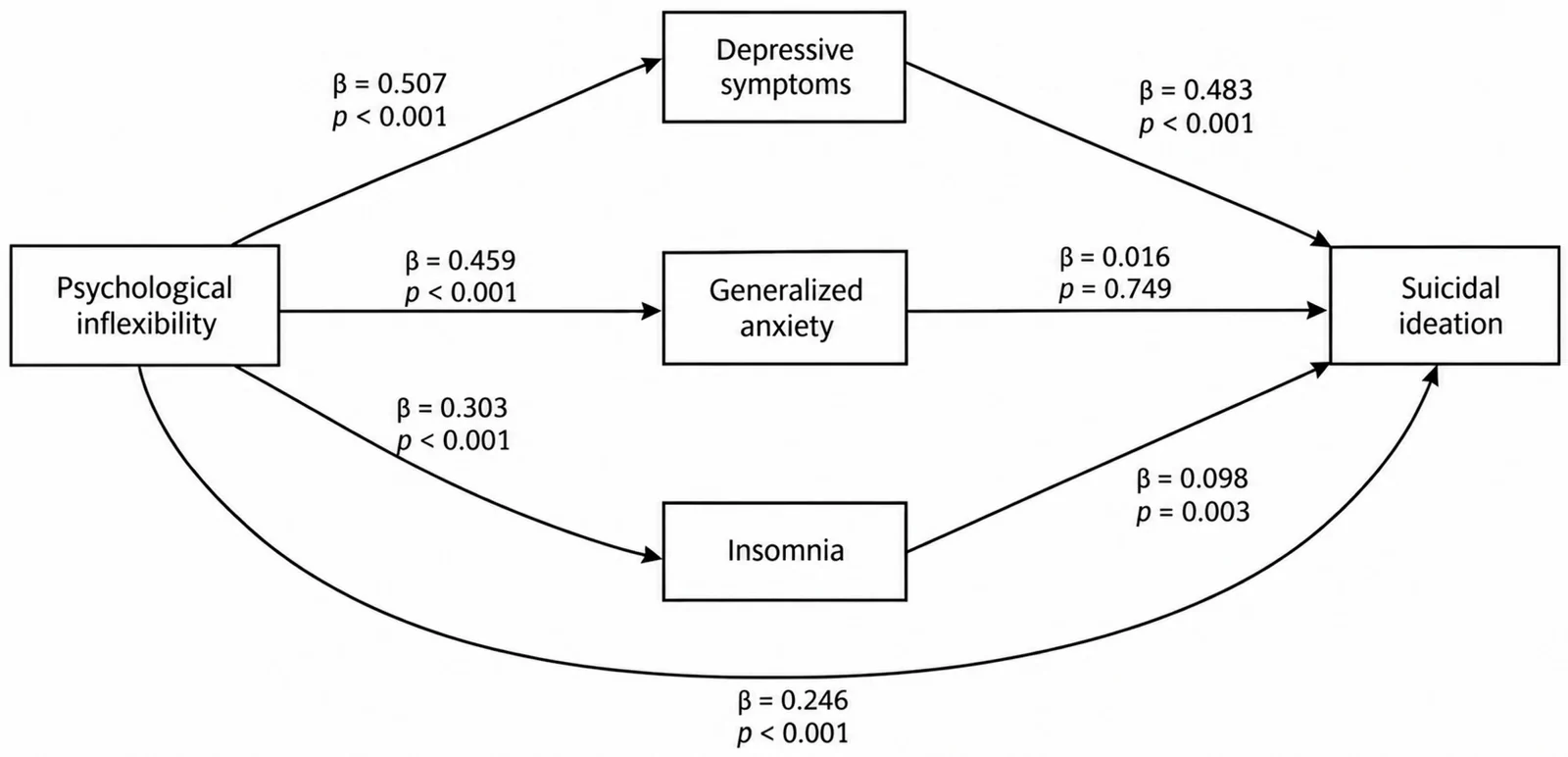
Multiple mediation model linking psychological inflexibility to suicidal ideation through depressive symptoms, generalized anxiety, and insomnia. Note. Values shown on the paths are standardized regression coefficients (β) with their corresponding *p*-values. The five constructs were modeled as latent variables using their respective item-level indicators; observed indicators, factor loadings, residual terms, and residual covariances among the mediators are omitted for visual clarity.

**Table 1:** Sociodemographic and pregnancy-related characteristics of the participants.

	*n* (%)
**Age**	30.97 ± 6.68
**Employment**	
Yes	321 (33.4%)
No	641 (66.6%)
**Marital status**	
Single	613 (63.7%)
Married	330 (34.3%)
Divorced	15 (1.6%)
Widowed	4 (0.4%)
**Area of residence**	
Urban	821 (85.3%)
Rural	141 (14.7%)
**Education level**	
No formal education	3 (0.3%)
Incomplete primary	13 (1.4%)
Completed primary	7 (0.7%)
Incomplete secondary	94 (9.8%)
Completed secondary	335 (34.8%)
Incomplete technical	68 (7.1%)
Completed technical	165 (17.2%)
Incomplete university	148 (15.4%)
Completed university	118 (12.3%)
Incomplete postgraduate	6 (0.6%)
Completed postgraduate	5 (0.5%)
**Gestational stage**	
First trimester (1–3 months)	188 (19.5%)
Second trimester (4–6 months)	364 (37.8%)
Third trimester (7+ months)	410 (42.6%)
**Planned pregnancy**	
Yes	483 (50.2%)
No	479 (49.8%)
**Perceived partner rejection of the current pregnancy**	
Never	736 (76.5%)
Rarely	131 (13.6%)
Sometimes	59 (6.1%)
Often	18 (1.9%)
Almost every day	18 (1.9%)
**Suicidal ideation**	
Yes	53 (5.5%)
No	909 (94.5%)
**Suicide attempt**	
Yes	11 (1.1%)
No	951 (98.9%)
**History of abortion**	
None	512 (53.2%)
Spontaneous abortion	362 (37.6%)
Therapeutic induced abortion	41 (4.3%)
Voluntary induced abortion	47 (4.9%)
**Current abortion intention**	
Yes	33 (3.4%)
No	929 (96.6%)
**Attitude toward pregnancy**	
Very negative	10 (1%)
Negative	12 (1.2%)
Neutral	131 (13.6%)
Positive	503 (52.3%)
Very positive	306 (31.8%)

**Table 2:** Factor loadings of the measurement component of the structural equation model.

Latent Variable	Indicator	Estimate	SE	z	Standardized Loading (λ)
Psychological inflexibility	AAQ1	1.000 ^a^	-	-	0.706
	AAQ2	1.080	0.041	26.134	0.780
	AAQ3	1.095	0.045	24.525	0.769
	AAQ4	1.175	0.047	25.083	0.811
	AAQ5	1.218	0.048	25.452	0.826
	AAQ6	1.142	0.053	21.537	0.791
	AAQ7	1.186	0.049	24.112	0.827
Depressive symptoms	PHQ1	1.000 ^a^	-	-	0.701
	PHQ2	1.170	0.060	19.465	0.925
Generalized anxiety	GAD1	1.000 ^a^	-	-	0.854
	GAD2	1.055	0.044	24.009	0.892
Insomnia	EIA1	1.000 ^a^	-	-	0.697
	EIA2	1.039	0.035	29.909	0.754
	EIA3	1.000	0.046	21.621	0.764
	EIA4	1.250	0.056	22.223	0.868
	EIA5	1.250	0.059	21.359	0.860
Suicidal ideation	FSII1	1.000 ^a^	-	-	0.887
	FSII2	1.120	0.039	28.547	0.850
	FSII3	1.173	0.049	24.123	0.847
	FSII4	1.141	0.047	24.266	0.921
	FSII5	1.271	0.059	21.615	0.910

Note. Estimate = unstandardized factor loading; SE = standard error; λ = standardized factor loading. All freely estimated factor loadings were statistically significant at *p* < 0.001. ^a^ The loading of the first indicator of each latent variable was fixed to 1.00 to establish the metric of the factor. The PHQ-2 and GAD-2 were specified as two-indicator latent variables using their individual items rather than their total scores.

**Table 3:** Direct, indirect, and total effects of psychological inflexibility on suicidal ideation through depressive symptoms, generalized anxiety, and insomnia.

Type	Effects	Estimate	Robust SE	95% CI	β	z	*p*
Structural paths	PI → DS	0.301	0.027	[0.248, 0.354]	0.507	11.057	0.001
	PI → GA	0.321	0.028	[0.267, 0.376]	0.459	11.553	0.001
	PI → INS	0.177	0.023	[0.131, 0.222]	0.303	7.620	0.001
	DS → SI	0.400	0.057	[0.289, 0.511]	0.483	7.036	0.001
	GA → SI	0.011	0.034	[−0.056, 0.078]	0.016	0.319	0.749
	INS → SI	0.083	0.028	[0.028, 0.138]	0.098	2.944	0.003
Direct effect	PI → SI	0.121	0.021	[0.080, 0.162]	0.246	5.836	0.001
Indirect effects	PI → DS → SI	0.120	0.021	[0.080, 0.161]	0.245	5.828	0.001
	PI → GA → SI	0.004	0.011	[−0.018, 0.025]	0.007	0.320	0.749
	PI → INS → SI	0.015	0.005	[0.004, 0.025]	0.030	2.716	0.007
	Total indirect effect	0.139	0.020	[0.100, 0.177]	0.282	7.034	0.001
Total effect	PI → SI	0.259	0.028	[0.204, 0.315]	0.528	9.137	0.001

*Note*. PI = Psychological inflexibility; DSs = Depressive symptoms; GA = Generalized anxiety; INS = Insomnia; SI = Suicidal ideation; Robust SE = robust standard error; CI = confidence interval; β = standardized regression coefficient. The 95% confidence intervals correspond to the unstandardized estimates.

## Data Availability

The data presented in this study are available upon request from the corresponding author due to privacy issues.
